# Strong immunogenicity and protection against SARS-CoV-2 in hamsters induced by heterologous boost vaccination with an MVA-based COVID-19 vaccine candidate

**DOI:** 10.1099/jgv.0.002180

**Published:** 2025-11-19

**Authors:** Sonja Ohrnberger, Christian Meyer zu Natrup, Sabrina Clever, Lisa-Marie Schünemann, Federico Armando, Malgorzata Ciurkiewicz, Wolfgang Baumgärtner, Georgia Kalodimou, Gerd Sutter, Alina Tscherne, Asisa Volz

**Affiliations:** 1 Institute of Virology, University of Veterinary Medicine Hannover, 30559 Hanover, Germany; 2 Department of Pathology, University of Veterinary Medicine Hannover, 30559 Hanover, Germany; 3 Division of Virology, Department of Veterinary Sciences, Ludwig Maximilians University (LMU Munich), 85764 Oberschleißheim, Germany

**Keywords:** emerging viruses, heterologous vaccination, low-dose vaccination, modified vaccinia virus Ankara, severe acute respiratory syndrome coronavirus 2 (SARS-CoV-2)

## Abstract

Over the last decade, heterologous prime–boost vaccination regimens have been established as a promising strategy to enhance immune responses and make optimal use of the advantages of different vaccine platforms. Modified vaccinia virus Ankara (MVA), a replication-deficient poxviral vector with an established safety profile, is under clinical investigation as a versatile recombinant vaccine platform against various infectious diseases. In the context of coronavirus disease 2019 (COVID-19), a recombinant MVA-based vaccine candidate expressing the prefusion-stabilized severe acute respiratory syndrome coronavirus 2 (SARS-CoV-2) spike protein (MVA-ST) has demonstrated safety, immunogenicity and protection in preclinical studies using different animal models. Furthermore, a phase Ib clinical trial in healthy adults showed that MVA-ST is safe, well-tolerated and immunogenic when used as a booster following mRNA priming. In this study, we evaluated heterologous prime–boost vaccination regimens using MVA-ST as a booster in Syrian hamsters. Hamsters were primed with an mRNA vaccine (BNT162b2, BioNTech/Pfizer) or the adenoviral vector vaccine Ad26.COV2.S (Janssen) and subsequently boosted with MVA-ST at a dose of 10⁸ p.f.u. These heterologous vaccination regimens induced robust protection against severe SARS-CoV-2 disease, with superior immunogenicity compared to homologous MVA-ST vaccination. Notably, even a lower booster dose (10⁷ p.f.u.) of MVA-ST following mRNA priming conferred strong protection against SARS-CoV-2 challenge infection, while still associated with limited viral shedding from the upper respiratory tract. These findings highlight the potential of MVA-ST as a heterologous booster to enhance the immunogenicity and protective efficacy of existing COVID-19 vaccines and also to improve vaccination strategies against other emerging pathogens.

## Introduction

In 2019, the emergence of severe acute respiratory syndrome coronavirus 2 (SARS-CoV-2), the causative agent of coronavirus disease 2019 (COVID-19), resulted in a global pandemic. Several licensed COVID-19 vaccines have been rapidly developed and are now available using both established and novel technological platforms [1]. Among the most widely deployed were mRNA-based vaccines, such as BNT162b2 (BioNTech/Pfizer) and mRNA-1273 (Moderna). They both deliver lipid-nanoparticle-encapsulated messenger RNA, encoding a prefusion-stabilized form of the SARS-CoV-2 spike (S) protein [2,3]. Even though mRNA vaccines had not been licensed for use in humans prior to the COVID-19 pandemic, they demonstrated safety and efficacy across diverse populations upon deployment [4]. Another vaccine platform used during the COVID-19 pandemic is non-replicating adenoviruses as a well-established viral vector platform for many decades. ChAdOx1 nCoV-19 (AstraZeneca/Oxford) expresses the native spike protein, generally associated with the induction of lower titres of neutralizing antibodies following vaccination [5]. In contrast, the Ad26.COV.2.S (Janssen) expresses the prefusion-stabilized version of the S protein and has been licensed for a single-shot vaccination approach [6]. All four vaccines are highly immunogenic, inducing both a robust systemic humoral and cellular immune response, characterized by humoral and cellular activation [7]. The exceptional circumstances of the pandemic enabled an unprecedented rapid development and approval of vaccines but have also been associated with several initial hurdles. This included limited large-scale manufacturing capacities, logistical challenges and disparities in vaccine allocation and deployment. Specifically, mRNA vaccines were reliant on stringent cold-chain maintenance, limiting their use in low-resource settings [8]. The nationwide vaccination campaigns also brought the accumulation of rare but serious adverse effects to light. Vaccine-induced immune thrombotic thrombocytopenia could be associated with ChAdOx1 nCoV-19 (AstraZeneca/Oxford) administration. This led to reduced popularity and raised concerns regarding the safety of booster injections, as required by the two-dose vaccination regimen [9]. In combination with concerns about pre-existing vector immunity, a known issue for adenoviral platforms, it emphasized the need for alternative and innovative vaccination strategies [10].

Now that a substantial proportion of the global population is already vaccinated, the perceived threat of the pandemic has diminished, and public concern has noticeably declined. However, the continued emergence of new SARS-CoV-2 variants in combination with fading immunity and discontinued vaccination might pave the way for new outbreak scenarios. In this context, heterologous vaccination strategies can provide a suitable alternative to homologous vaccination to balance production demand and distribution. In addition, heterologous prime–boost vaccination approaches might be a promising alternative to classical homologous vaccination regimens, as they are generally associated with more robust immune responses and reduced anti-vector immunity [11,12]. Thus, it is crucial to evaluate outcomes of heterologous vaccination strategies with regard to protection, immunogenicity and long-term impact.

The modified vaccinia virus Ankara (MVA), a highly attenuated and replication-deficient vaccinia virus, is already licensed as a third-generation smallpox and mpox vaccine [13,14]. MVA has been extensively studied as a non-replicating, versatile viral vector for vaccines targeting a range of infectious diseases and some cancers [15]. As such, MVA represents a promising platform for developing candidate vaccines that elicit robust innate and adaptive immune responses. Recently, we developed an MVA-based vaccine against SARS-CoV-2 expressing the prefusion-stabilized S protein (MVA-ST). MVA-ST showed promising results in preclinical and clinical studies [16,17]. While repeated application of MVA-based vaccines enhanced immune responses without indication of anti-vector immunity, it is considered to further facilitate the immunogenicity of other vaccines when administered in a heterologous schedule [18]. This can be seen for the licensed vaccine against the Ebola virus (EBOV), combining the adenovirus-based vaccine Zabdeno (Ad26.ZEBOV) with Mvabea (MVA-BN-Filo) [19]. Moreover, a heterologous prime–boost vaccination approach has also been evaluated with the MVA-SARS-2-ST in a phase Ia/b clinical study in humans. Here, Mayer *et al*. evaluated the effects of homologous and heterologous approaches comparing MVA-, mRNA- and adenovirus-based vaccines. MVA-SARS-2-ST in a homologous approach generally elicited comparable or lower immune responses as prime–boost with an mRNA vaccine or adenovirus-based vaccines. In contrast, when administered as a third vaccination after mRNA prime–boost immunization, it improved subunit 1 (S1)-specific IgG antibody responses in individuals with low baseline values. This has not been seen for high baseline values. The S1 subunit contains the receptor-binding domain and has been found to present the main target for neutralizing antibodies. Therefore, neutralization capacity strongly correlates with levels of induced S1-specific IgG antibody responses. Of note, they also demonstrated the activation of substantial levels of cellular immune responses after MVA-SARS-2-ST boost [17]. These results indicated an advantage of heterologous prime–boost vaccination approaches using an MVA-ST boost for COVID-19 vaccinations. To further investigate the patterns of immune responses and protective efficacy of such heterologous prime–boost approaches, we used the Syrian hamster model for COVID-19 and tested the administration of 10^8^ p.f.u. MVA-ST boost vaccination after mRNA or adenovirus-based priming. Both heterologous prime–boost regimens provided strong protection against severe disease, associated with a robust activation of SARS-CoV-2-specific immune responses. Of note, a lower dose of 10^7^ p.f.u. MVA-ST was able to provide sufficient protection after mRNA prime but was associated with low levels of viral shedding in the upper respiratory tract. These data further support the use of MVA-based vaccines as a boost in heterologous vaccination regimens against COVID-19.

## Methods

### Vaccines

The generation and preclinical characterization of MVA-SARS-2-ST (MVA-ST), encoding a prefusion-stabilized S protein of the SARS-CoV-2 Wuhan Hu-1 (GenBank accession no. MN908947.1) with an inactivated S1/subunit 2 (S2) furin cleavage site, was described previously by Meyer Zu Natrup *et al.* [16]. BNT162b2 by BioNTech/Pfizer, here referred to as mRNA, is a licensed vaccine consisting of nucleoside-modified mRNA also encoding the prefusion-stabilized S protein, formulated in lipid nanoparticles [2]. The licensed Ad26.COV2.S vaccine by Janssen, here referred to as adenoviral vector (AdV), is based on a non-replicating adenovirus serotype 26 vector and also encodes a prefusion-stabilized SARS-CoV-2 S protein [6].

### Virus

SARS-CoV-2 (isolate Germany/BavPat1/2020, NR-52370; isolate hCoV-19/USA/PHC658/2021, lineage B.1.617.2 Delta variant, NR-55611; isolate hCoV-19/USA/MD-HP20874/2021, lineage B.1.1.529, Omicron variant, NR-56461), obtained from BEI Resources (NIAID, NIH), was propagated in VeroE6 cells (ATCC #CRL-1586) cultured in Dulbecco’s Modified Eagle Medium (DMEM; Sigma-Aldrich GmbH, St Louis, MO, USA) supplemented with 2% FBS, 1% penicillin–streptomycin and 1% l-glutamine (Capricorn Scientific GmbH, Ebsdorfergrund, Germany) at 37 °C.

### Ethical statement

All experiments were performed in accordance with the European and national regulations for animal experimentation (European Directive 2010/63/EU; Animal Welfare Acts in Germany) and Animal Welfare Act and approved by the Niedersächsisches Landesamt für Verbraucherschutz und Lebensmittelsicherheit (LAVES, Lower Saxony, Germany). Before the immunization, the hamsters (*Mesocricetus auratus*; breed RjHan:AURA), bought from the Janvier Labs (SAINT BERTHEVIN CEDEX, France), were allowed to adapt to the stables for at least 1 week. One week before the infection (42 days after the start of the study), all animals were transferred to a biosafety level (BSL)-3e laboratory and underwent 1 week of acclimatization before challenge infection. All animal and laboratory work with infectious SARS-CoV-2 was performed in a BSL-3e laboratory and facilities at the Research Center for Emerging Infections and Zoonoses, University of Veterinary Medicine, Hannover.

### Immunization experiments in Syrian hamsters

Groups of Syrian hamsters (*M. auratus*; breed RjHan:AURA) received a prime immunization of 12 µg mRNA (*n*=8) or 100 µl sterile PBS (*n*=4), followed by a low-dose (LD) MVA-ST booster of 1×10^7^ or 1×10^8^ p.f.u. empty-MVA-vector control (MVA) 21 days later. All injections were administered intramuscularly into the quadriceps muscle of the left hind leg. We closely observed and monitored the hamsters after the immunizations for adverse effects and weight loss. In a second experiment, groups of Syrian hamsters (*M. auratus*; breed RjHan:AURA; *n*=4–8) received different heterologous prime–boost immunizations over a 3-week interval. Either 1×10^9^ viral particle (VP) AdV, 12 µg mRNA or 100 µl sterile PBS was administered as prime immunization, followed by a high dose (HD) MVA-ST booster of 1×10^8^ p.f.u. or MVA 21 days later. Again, all injections were administered intramuscularly into the quadriceps muscle of the left hind leg, and we closely observed and monitored the hamsters after the immunizations for adverse effects and weight loss.

### SARS-CoV-2 infection in Syrian hamsters

For SARS-CoV-2 challenge infection, animals were kept in individually ventilated cages (Tecniplast, Buguggiate, Italy) under BSL-3 conditions in approved facilities of the University for Veterinary Medicine Hannover. Hamsters were challenged by intranasal infection under anaesthesia with 1×10^4^ TCID_50_ of SARS-CoV-2 (Isolate Germany/BavPat1/2020, NR-52370) received from BEI Resources (NIAID, NIH). Cardiovascular system, fur/skin condition, lower and upper respiratory tract, environmental and social behaviour/general condition/locomotion and neurological condition were monitored for symptoms associated with COVID-19 at least twice daily after respiratory infection and evaluated with a clinical scoring sheet. This included enhanced respiratory rate, scuffed or ruffled fur or reduced activity. Body weights were checked daily.

### TCID_50_

Nasal and oropharyngeal swabs from days 3 and 6 post-infection, as well as homogenized lung and brain tissue samples, were analysed with a TCID_50_ to determine the titres of infectious SARS-CoV-2. Samples were collected in 1 ml of DMEM (Sigma-Aldrich GmbH), and tissues were homogenized with the TissueLyser-II (Qiagen, Hilden, Germany) for downstream analysis. We performed tenfold serial dilutions of swabs and homogenized lung and brain samples in DMEM containing 5% FBS (Capricorn Scientific GmbH). Dilutions were plated in quadruplicate onto 96-well plates containing VeroE6 cells and incubated for 4 days at 37 °C. Titres were calculated in TCID_50_/ml, using the Reed–Muench method based on cytopathic effects in the cells as described in Meyer Zu Natrup *et al.* [16].

### Reverse transcription quantitative real-time PCR

To determine levels of viral RNA of SARS-CoV-2 in the swabs, lungs and brains, we performed reverse transcription quantitative real-time PCR (RT-qPCR), specified to target different SARS-CoV-2 genes. Briefly, RNA was isolated from swabs and homogenized brain and lung tissues with the KingFisher Flex and NucleoMag RNA kit (MACHEREY-NAGEL GmbH and Co. KG, Düren, Germany) according to the manufacturer’s protocol. We subsequently performed RT-qPCR using the Luna® Universal Probe One-Step RT-qPCR Kit (New England Biolabs GmbH, Frankfurt am Main, Germany) on a CFX96 Touch Real-Time PCR system (Bio-Rad, Feldkirchen, Germany). Ct values obtained for each sample were then compared against a standard curve, and corresponding viral RNA load was calculated in copy numbers/µl. RT-qPCR targeting the RNA-dependent RNA polymerase (RdRp) gene of SARS-CoV-2 was performed with SARS-2-IP4 primers (Table 1) and probe (5′-TCA TAC AAA CCA CGC CAG G-3′ [5′]FAM [3′]BHQ-1). RT-qPCR specific to the envelope € gene and subgenomic E (subE) of SARS-CoV-2 was performed with the respective primers (Table 1) in combination with the probe (5′-ACA CTA GCC ATC CTT ACT GCG CTT CG-3′[5′]FAM [3′]BHQ-1). Homogenates of lung tissue samples were analysed for the expression of different immune mediators. RT-qPCR was performed with the commercially available Luna® Universal One-Step RT-qPCR Kit (New England Biolabs GmbH) in the same system as mentioned above. We used primers specific to the hamster’s cytokines IL-6, IL-10, CCL5, CxCl11 and ISG15. *β*-Actin was used as a housekeeping gene with the corresponding forward and reverse primers (Table 1). The amount of respective cytokine relative to *β*-actin was calculated using the ∆Ct method and presented as normalized expression.

**Table 1.** Oligonucleotide sequences used for RT-qPCR analysis of SARS-CoV-2-specific genes and hamster-specific immune modulators. Shown are the gene targets and nucleotide sequences and primer direction

Target	Gene	Direction	Sequence
SARS-CoV-2 specific	RdRp	Forward Reverse	5′-GGT AAC TGG TAT GAT TTC G-3′ 5′-CTG GTC AAG GTT AAT ATA GG-3′
	E gene	Forward Reverse	5′-ACA GGT ACG TTA ATA GTT AAT AGC GT-3′ 5′-ATA TTG CAG CAG TAC GCA CAC A-3′
	Subgenomic E	Forward Reverse	5′-CGA TCT CTT GTA GAT CTG TTC TC-3′ 5′-ATA TTG CAG CAG TAC GCA CAC A-3′
Hamster specific	CCL5	Forward Reverse	5′-ACT GCC TCG TGT TCA CAT CA-3′ 5′-TTC GGG TGA CAA AAA CGA CT-3′
	CxCl11	Forward Reverse	5′-CCG CCT CAT ACG GGA AAT GT-3′ 5′-AAG ACA GAA GGT TGG GCT CG-3′
	IL-10	Forward Reverse	5′-GAA GGA CCA GCT GGA CAA CA-3′ 5′-TGG CAA CCC AAG TAA CCC TTA-3′
	IL-6	Forward Reverse	5’- GGT ATG CTA AGG CAC AGC ACA CT-3′ 5’- CCT GAA AGC ACT TGA AGA ATT CC-3′
	ISG15	Forward Reverse	5’- TCT ATG AGG TCC GGC TGA CA-3′ 5’- GCA CTG GGG CTT TAG GTC AT-3′
	*β*-Actin	Forward Reverse	5′-CCA AGG CCA ACC GTG AAA AG-3′ 5′-ATG GCT ACG TAC ATG GCT GG-3′

### Plaque reduction neutralization test 50

Blood was collected at different time points after the immunization (days 0, 21 and 42) and on day 6 post-infection. Serum was prepared by centrifugation of the coagulated blood at 1.300 ***g*** for 5 min. Serum samples were then stored at −80 °C until further use. Plaque reduction neutralization test 50 (PRNT_50_) assays were conducted to determine the titres of neutralizing antibodies against SARS-CoV-2 isolates BavPat1, Delta (lineage B.1.617.2) and Omicron (lineage B.1.1.529) in serum samples. Serum was heat inactivated for 30 min at 56 °C, diluted in twofold serial dilutions and plated in duplicates on 96-well plates. Subsequently, 50 µl of the respective SARS-CoV-2 isolate, corresponding to 600 TCID_50_, was added to each well and incubated at 37 °C for 1 h. The virus-serum mixtures were then transferred onto 96-well plates containing VeroE6 cells and incubated for 45 min. A 1 : 1 mixture of DMEM and Avicel RC-591 (DuPont, Nutrition and Biosciences, Lyngby, Denmark) was added as an overlay with 100 µL per well and plates were further incubated for an additional 24 h at 37 °C. To inactivate SARS-CoV-2, cells were fixed with 4% formaldehyde in PBS prior to staining. For detection, a polyclonal rabbit antibody against the SARS-CoV-2 nucleoprotein (clone 40588-T62, Sino Biological, Wayne, PA, USA) was used, followed by a peroxidase-conjugated goat anti-rabbit IgG secondary antibody (Agilent Dako, Glostrup, Denmark). Signal development was achieved using TrueBlue™ Peroxidase Substrate (SeraCare, Milford, MA, USA). Infected cell foci were quantified and analysed using the ImmunoSpot® reader (CTL Europe GmbH, Bonn, Germany). PRNT_50_ titre was defined as the reciprocal of the highest serum dilution resulting in a reduction of more than 50% in SARS-CoV-2 plaque formation.

### Enzyme-linked ImmunoSpot

Hamsters were sacrificed 6 days post-infection (dpi) (54 days after start of the study), and spleens were collected to immediately isolate splenocytes as previously described [20]. Briefly, spleens were grated through a 70 µm cell strainer (Falcon®, Sigma-Aldrich, Taufkirchen, Germany) and washed with RPMI-10 (RPMI 1640 medium containing 10% FBS, 1% penicillin–streptomycin and 1% HEPES; Sigma-Aldrich). Red blood cells were lysed with Red Blood Cell Lysis Buffer (Sigma-Aldrich) and splenocytes were counted with the MACSQuant (Miltenyi Biotec B.V. and Co., KG, Bergisch Gladbach, Germany). 3×10^5^ splenocytes per well were seeded onto 96-well round bottom plates. Overlapping peptide pools specific to the S1 and S2 of SARS-CoV-2 were acquired from JPT Peptide Technologies (Berlin, Germany) and a peptide pool specific to the SARS-CoV-2 nucleocapsid (N) from BEI Resources (NIAID, NIH). One hundred microlitres of peptides was added to the splenocytes at a concentration of 1 µg peptide/ml, diluted in RPMI-10, for stimulation. Cells stimulated with phorbol myristate acetate and ionomycin (Sigma-Aldrich) and non-stimulated cells served as positive and negative controls, respectively. PVDF membrane plates (Merck Millipore, Burlington, MA, USA) were coated with hamster-specific anti-IFN-*γ* monoclonal antibody (Mabtech, Nacka, Sweden). Splenocytes and stimulants were transferred from the 96-well round-bottom plates onto the coated PVDF membrane plates and incubated for 36 h at 37 °C. The splenocyte/peptide mixture was removed, and staining was conducted according to the manufacturer’s instructions. IFN-γ spot-forming cells (SFCs) were quantified with the automated enzyme-linked ImmunoSpot (ELISpot) Reader ImmunoSpot 7.0.20.1 software (ImmunoSpot, Bonn, Germany), and numbers were calculated in relation to 1×10^6^ splenocytes.

### Histopathology

On the day of necropsy, the entire left lung lobes were collected and prepared for histopathological analysis by fixation with 10% formalin followed by embedding in paraffin. Sections of 2–3 micron-thick slices were generated and stained with haematoxylin and eosin (HE) to evaluate lung lesions. More specifically, we scored alveolar lesions based on inflammation, regeneration, necrosis/desquamation and loss of alveolar cells, atypical large/syncytial cells, intra-alveolar fibrin, alveolar oedema and haemorrhage. Airway lesions were assessed based on inflammation, necrosis and hyperplasia and vascular lesions on vasculitis, perivascular cuffing, oedema and haemorrhage. The total sum of alveolar, airway and vascular lesions score was calculated to further reflect the severity of findings in the respective lung anatomical compartments. Details on the scoring system were described previously [21].

### Statistical analysis

Statistical analysis was conducted using GraphPad Prism 10.5.0 (GraphPad Software Inc.). Multiple data sets were analysed by the Kruskal–Wallis test combined with Dunn’s multiple comparisons test. Two data sets were compared with the Kolmogorov–Smirnov test. Data are presented as median values with interquartile range. The asterisks represent statistically significant differences between two groups: *P<0.05, **P<0.01, ***P<0.001 and ****P<0,0001. Exact *P*-values are shown for comparisons with a trend toward significance (0.05≤*P*<0.10). Non-significant differences outside this range are not indicated.

## Results

### LD MVA-ST booster vaccination protects hamsters from clinically manifested disease

**Fig. 1.**
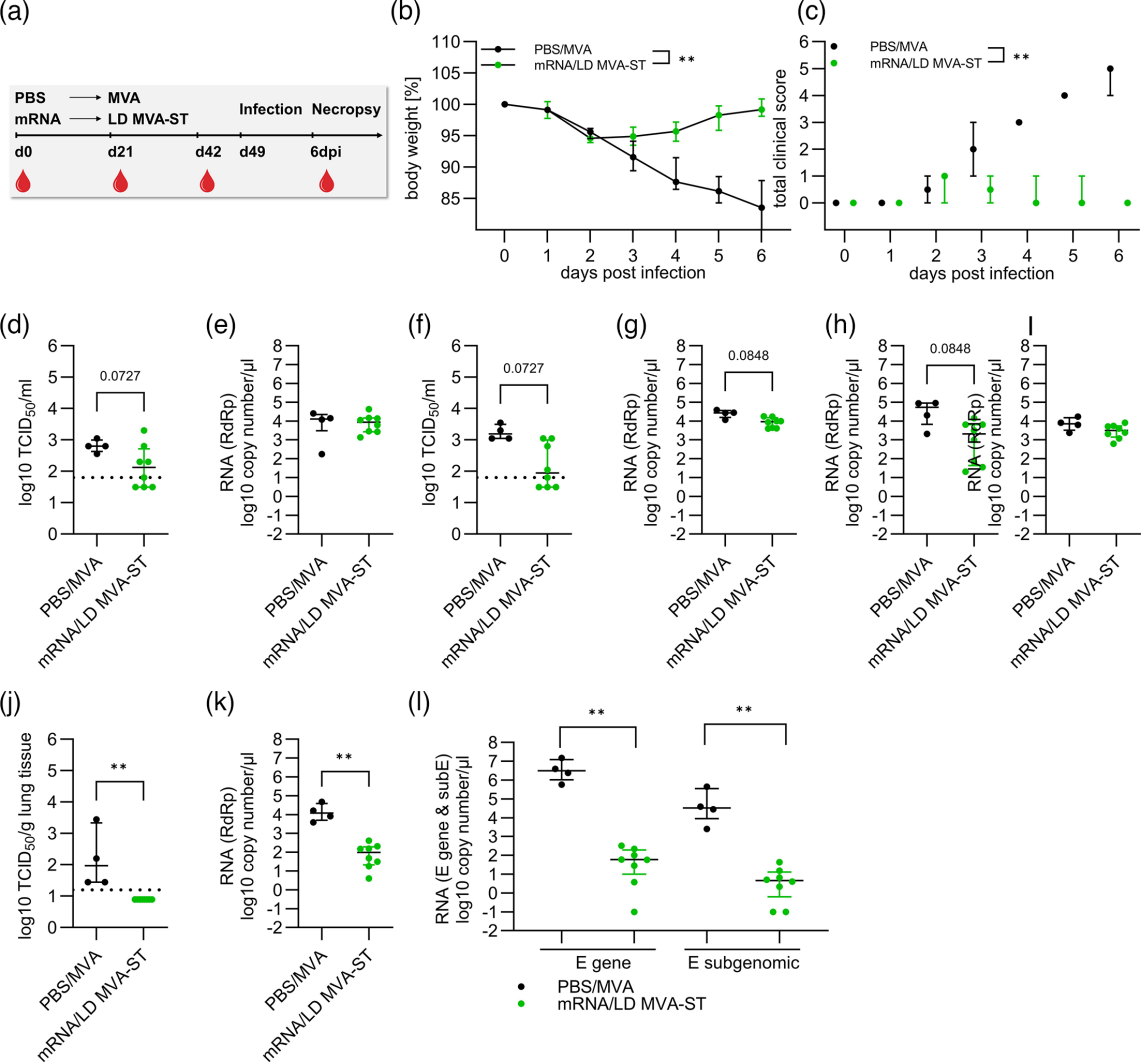
LD MVA-ST booster prevents clinical disease in hamsters. (**a**) Groups of hamsters (*n*=4 for PBS/MVA, *n*=8 for mRNA/LD MVA-ST) were immunized first with a dose of 12 µg mRNA and boosted 21 days later with 10^7^ p.f.u. MVA-ST using the intramuscular route. Twenty-eight days later, all animals were challenged with 1×10^4^ TCID_50_ SARS-CoV-2 BavPat1 isolate via the intranasal route. (**b**) Body weight was monitored daily. (**c**) Clinical condition was assessed using the clinical scoring system described in the Methods section. (**d, e**) Nasal and (**f, g**) oropharyngeal swabs collected on day 3 post-challenge were analysed for titres of infectious SARS-CoV-2 using TCID_50_ per gramme lung tissue and copies of genomic RNA by RdRp qRT-PCR. (**h**) Nasal and (**i**) oropharyngeal swabs collected on day 6 post-challenge were analysed for copies of genomic RNA by RdRp qRT-PCR. (**j–m**) Lung tissues were harvested on day 6 post-challenge and analysed for (**j**) infectious SARS-CoV-2 titres (TCID_50_ per gramme lung tissue) and copies of genomic RNA by (**k**) RdRp qRT-PCR and (**l**) E-gene qRT-PCR and subE qRT-PCR. Differences between the groups were analysed, determining the area under the curve (AUC) (**b, c**) prior to analysis with the Kolmogorov–Smirnov test (**b–f**). Data are presented as median values with interquartile range. The asterisks represent statistically significant differences between two groups: **P<0.01. Exact *P*-values are shown for comparisons with a trend toward significance (0.05≤*P*<0.10). Non-significant differences outside this range are not indicated.

Syrian hamsters were vaccinated in a heterologous prime–boost immunization schedule, receiving 12 µg mRNA, which corresponds to the standard dose in humans [22] via intramuscular injection into the left hind leg on day 0 followed by an LD booster injection of 1×10^7^ p.f.u. of MVA-ST on day 21. Hamsters primed with 100 µl sterile PBS and boosted with 1×10^8^ p.f.u. of empty MVA vector served as control (Fig. 1a). No adverse effects to the vaccinations were observed, and body weight was generally increasing over the 42-day observation period (Fig. S1A, available in the online Supplementary Material).

On day 49 post-vaccination, all hamsters were intranasally infected with 1×10^4^ TCID_50_ SARS-CoV-2 (BavPat1). All hamsters lost ~5% of their body weight, starting 2 dpi. The PBS/MVA-vaccinated control hamsters continued to show progressive weight loss until 6 dpi, losing ~15% of their original body weight, as measured on the day of the challenge. In contrast, no further loss of body weight was identified for the mRNA/LD MVA-ST–vaccinated hamsters (Fig. 1b).

Animals in the control group developed obvious clinical symptoms with enhanced breathing, scruffy fur and reduced activity, culminating on day 6 post-infection, with a total clinical score of 5. Due to mild weight loss in mRNA/LD MVA-ST–vaccinated hamsters, clinical scores of 1 were observed over days 2–4 post-infection (Fig. 1c).

At 3 dpi, levels of infectious SARS-CoV-2 were detected in the nasal swabs of the control-vaccinated hamsters with a median of 6.3×10^2^ TCID_50_. Lower titres were detected in the mRNA/LD MVA-ST–vaccinated hamsters with a median of 1.3×10^2^ TCID_50_ (Fig. 1d). RT-qPCR analysis targeting the RdRp gene of SARS-CoV-2 reached median titres of 1.3×10^4^ RNA copy numbers/μl for nasal swabs taken on 3 dpi in the PBS/MVA group. mRNA/LD MVA-ST–vaccinated hamsters mounted slightly lower levels in nasal swabs with a median of 8.7×10^3^ RNA copy numbers/μl (Fig. 1e).

This was also seen for oropharyngeal swabs taken on the same day for infectious SARS-CoV-2 titres (median of 1.6×10^3^ TCID_50_ for PBS/MVA, median 8.8×10^1^ TCID_50_ for mRNA/LD MVA-ST; Fig. 1f). On the RNA level, we detected RdRp copy numbers with a median of 2.8×10^4^ RNA copy numbers/μl for PBS/MVA-vaccinated hamsters and a median of 9.2×10^3^ RNA copy numbers/μl for the mRNA/LD MVA-ST–vaccinated hamsters (Fig. 1g).

All animals were euthanized at six dpi. Blood, nasal and oropharyngeal swabs and organ samples from lung, spleen and brain were collected for further analysis.

RT-qPCR of nasal swabs 6 dpi presented with a median of 5.3×10^4^ RNA copy numbers/μl in the PBS/MVA group. mRNA/LD MVA-ST–vaccinated hamsters showed lower values (median 2.1×10^3^ RNA copy numbers/μl; Fig. 1h). In oropharyngeal swabs taken 6 dpi, PBS/MVA control hamsters mounted a median of 7.2×10^3^ RNA copy numbers/μl, and mRNA/LD MVA-ST–vaccinated hamsters demonstrated a slightly lower median of 3.2×10^3^ RNA copy numbers/μl (Fig. 1i).

In the lungs of PBS/MVA control hamsters, we detected increased titres of infectious SARS-CoV-2 (median 9.3×10^1^ TCID_50_). In contrast, we did not detect titres of infectious SARS-CoV-2 in the lungs of the mRNA/LD MVA-ST–immunized hamsters (Fig. 1j).

RdRp in the lung presented with high levels in the PBS/MVA animals (median 1.2×10^5^ RNA copy numbers/μl) and significantly reduced levels in the mRNA/LD MVA-ST animals (median 9.8×10^1^ RNA copy numbers/μl; Fig. 1k). RT-qPCR targeting the E- and subE revealed significantly lower levels in the mRNA/LD MVA-ST–vaccinated animals (median 5.9×10^1^ RNA copy numbers/μl for E and median 4.6×10^0^ RNA copy numbers/μl for subE) compared to hamsters of the control group (median 3.2×10^6^ RNA copy numbers/μl for E and median 3.3×10^4^ RNA copy numbers/μl for subE; Fig. 1l).

RdRp levels were also evaluated in brain tissue samples, reaching significantly higher copy numbers in the PBS/MVA control animals (median 6.2×10^1^ RNA copy numbers/μl) compared to the mRNA/LD MVA-ST–vaccinated animals (median 3.1×10^0^ RNA copy numbers/μl; Fig. S1B). Similar results were observed for the E gene (median 1.0×10^2^ RNA copy numbers/μl for PBS/MVA and 2.3×10^1^ RNA copy numbers/μl for mRNA/LD MVA-ST). SubE titres were high in the PBS/MVA control animals (median 5.9×10^1^ RNA copy numbers/μl) and not detectable in mRNA/LD MVA-ST–vaccinated animals (Fig. S1C).

### LD MVA-ST boost induced the activation of SARS-CoV-2-specific neutralizing antibodies and IFN-*γ*-producing T cells

**Fig. 2.**
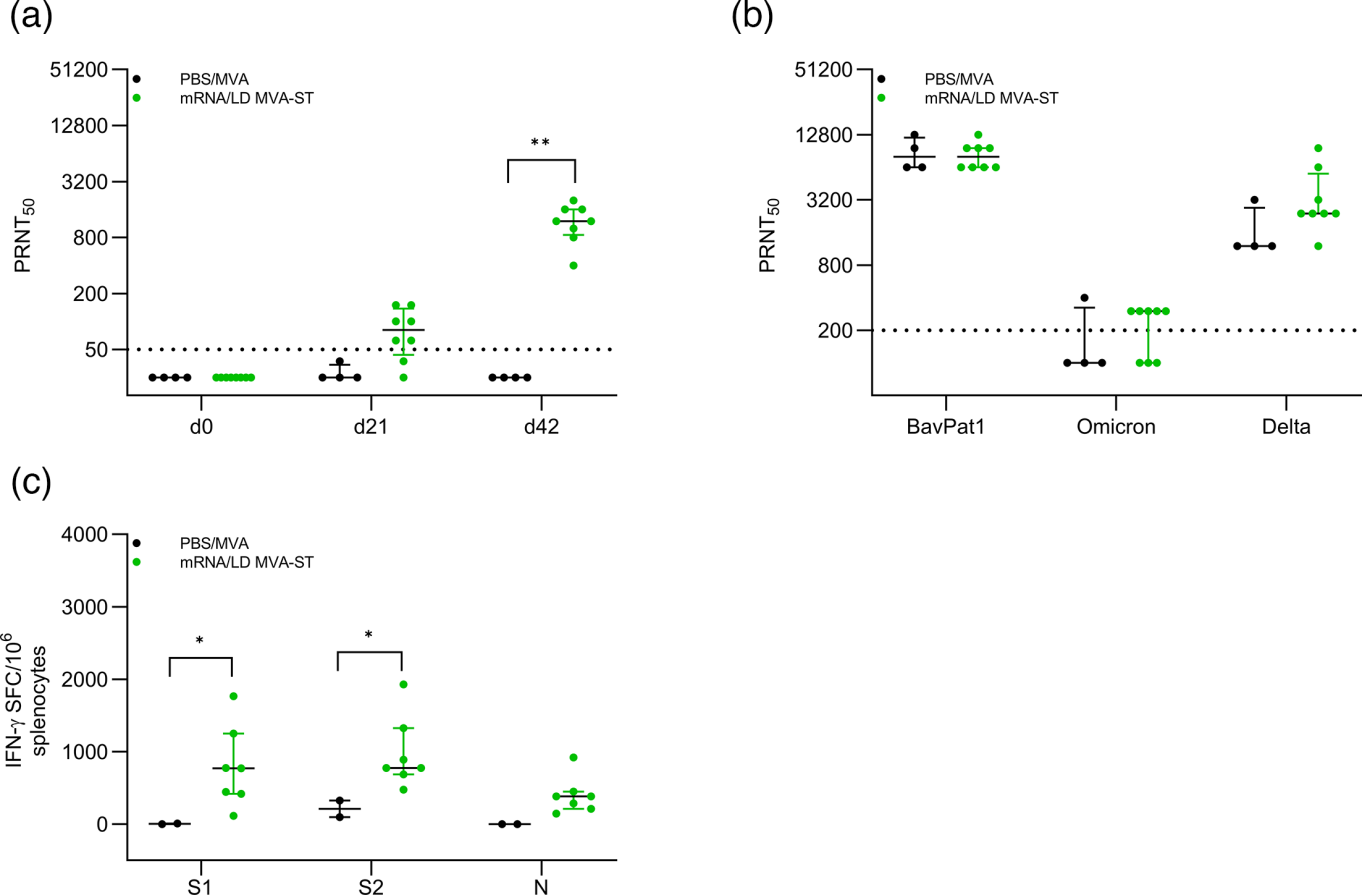
LD MVA-ST booster induces SARS-CoV-2-specific antibody and T cell responses. (**a**) Sera from mRNA/LD MVA-ST–vaccinated hamsters were prepared on days 0, 21 and 42 after vaccination and analysed for SARS-CoV-2 BavPat1-specific neutralizing antibodies by PRNT_50_. (**b**) Sera from day 6 after challenge infection were evaluated for neutralizing antibodies against three different SARS-CoV-2 variants: BavPat1, Omicron and Delta. (**c**) Splenocytes were collected and prepared on day 6 after SARS-CoV-2 BaVPat1 infection and stimulated with pools of overlapping peptides comprising the S1 and S2 subunit of SARS-CoV-2 S and N proteins and tested using the ELISpot assay. Differences between the groups were analysed with the Kolmogorov–Smirnov test (**a–c**). Dotted lines indicate the respective detection limit for PRNT_50_ (**a, b**). Data are presented as median values with interquartile range. Data are presented as median values with interquartile range. The asterisks represent statistically significant differences between two groups: *P<0.05 and **P<0.01. Exact *P*-values are shown for comparisons with a trend toward significance (0.05≤*P*<0.10). Non-significant differences outside this range are not indicated.

Hamsters of the PBS/MVA control group mounted no titres of neutralizing antibodies before infection, while vaccinated hamsters mounted detectable levels after the mRNA prime (median 1 : 81 PRNT_50_) and increased titres after the LD MVA-ST boost (median 1 : 1.200 PRNT_50_; Fig. 2a). After infection, both groups mounted similar levels of neutralizing antibodies against SARS-CoV-2 isolate BavPat1 (median 1 : 8.000 PRNT_50_ for both). mRNA/LD MVA-ST–vaccinated hamsters also mounted titres of neutralizing antibodies against the highly contagious SARS-CoV-2 Omicron variant (B.1.1.529, median 1 : 300 PRNT_50_), while titres in the control group remained below the detection limit. Hamsters vaccinated with mRNA/LD MVA-ST demonstrated significant levels of neutralizing antibodies against the SARS-CoV-2 Delta variant (B.1.617.2), achieving a median titre of 1 : 2.400 PRNT_50_, compared to the control group, which had a median titre of 1 : 1.200 PRNT_50_ (Fig. 2b). To evaluate the SARS-CoV-2-specific cellular immune responses, we monitored S1-, S2- and N-specific T cells *ex vivo* using ELISpot analysis of isolated and re-stimulated splenocytes at 6 dpi. Hamsters of the control group mounted only marginal numbers of IFN-*γ*-producing cells after stimulation with S1- (median 9.7 SFC/10^6^ splenocytes) and higher numbers after stimulation with S2- (median 328 SFC/10^6^ splenocytes) specific peptides. No IFN-γ-producing cells could be detected in PBS/MVA animals upon stimulation with N-specific peptides. mRNA/LD MVA-ST–vaccinated hamsters mounted higher numbers after stimulation with S1-, S2- and N-specific peptides (median 772 SFC/10^6^ splenocytes for S1, median 775 SFC/10^6^ splenocytes for S2 and median 383 SFC/10^6^ splenocytes for N; Fig. 2c).

### Pathohistological assessment of LD MVA-ST booster vaccination

**Fig. 3.**
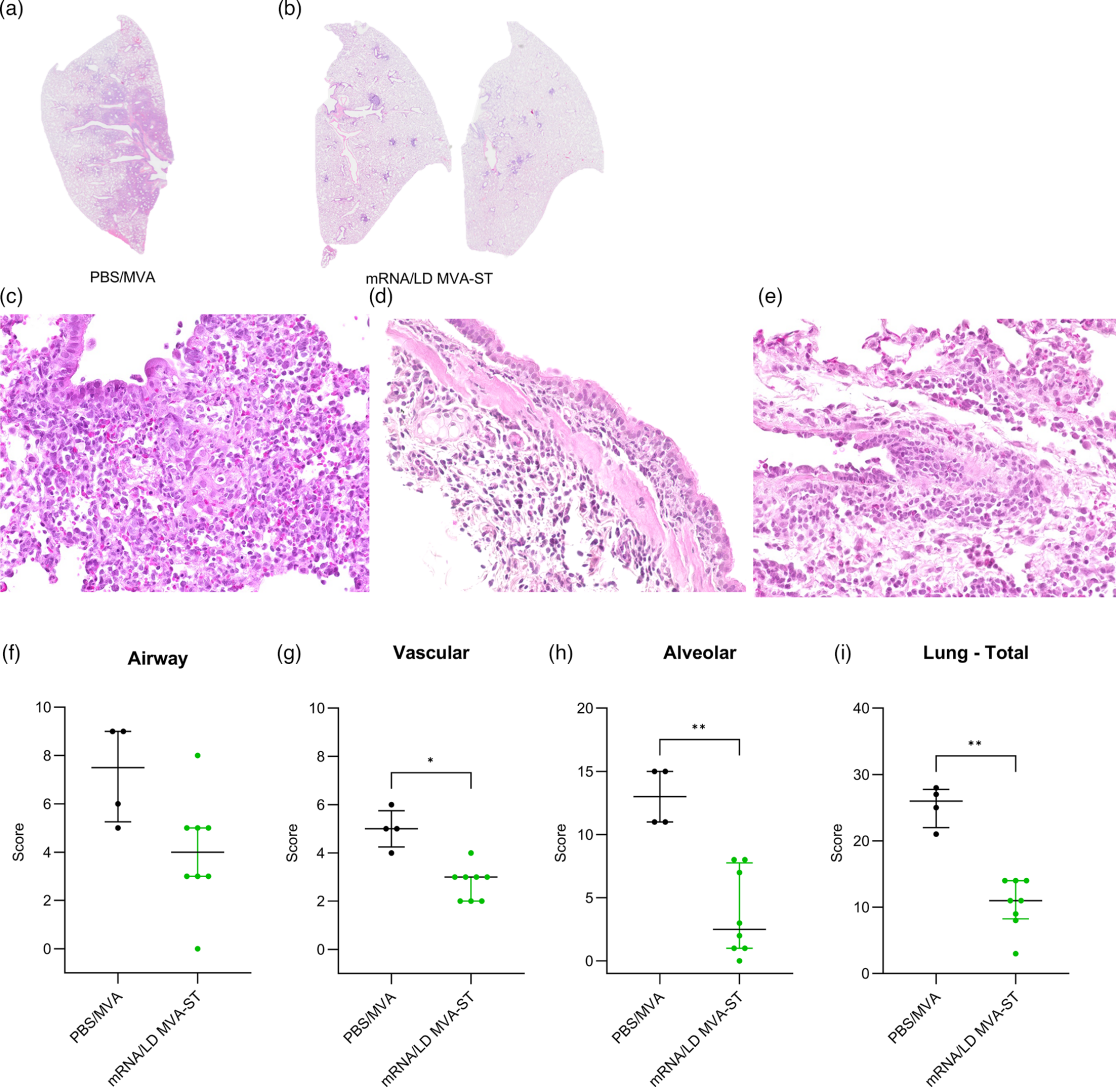
Histopathological lesions in the lungs of SARS-CoV-2 BavPat1–challenged hamsters vaccinated with PBS/MVA or mRNA/LD MVA-ST. (**a**) Representative overview images of HE-stained lung sections. Lung sections from PBS/MVA control–vaccinated animals show extensive areas of alveolar consolidation with markedly thickened alveolar septae and inflammatory infiltrates, while (**b**) mRNA/LD MVA-ST–vaccinated animals show no alveolar lesions. Higher magnification (**c**) of alveoli showing intraluminal and septal infiltrates, expanding to the terminal bronchioles, mainly composed of macrophages and neutrophils, (**d**) of a bronchus with intra- and subepithelial infiltrates, composed of lymphocytes and macrophages with single cell death events within the bronchial epithelium and (**e**) of a vessel with endothelialitis, endothelial cell hypertrophy, mural and perivascular infiltrates composed of macrophages and neutrophils. (**f–i**) Quantification of histopathological lesions by semiquantitative scoring of (**f**) airway lesions score, (**g**) vascular lesions, (**h**) alveolar lesions and (**i**) total scoring of the HE Lung. Differences between the groups were analysed with the Kolmogorov–Smirnov test (**f–i**). Data are presented as median values with interquartile range. The asterisks represent statistically significant differences between two groups: *P<0.05 and **P<0.01. Exact *P*-values are shown for comparisons with a trend toward significance (0.05≤*P*<0.10). Non-significant differences outside this range are not indicated.

To further characterize the protective efficacy of the heterologous prime–boost vaccination regimen, pathohistological evaluation was performed. The entire left lung lobes, taken at necropsy (6 dpi), were stained with HE and histologically evaluated. PBS/MVA control hamsters presented large areas of lung consolidation. Alveolar inflammation was marked by the accumulation of neutrophils and mononuclear cells, resulting in thickened alveolar septa and the obstruction of alveolar lumina (Fig. 3a). This was accompanied by alveolar epithelial necrosis, fibrin deposition and pronounced hyperplasia of type II pneumocytes (Fig. 3c). Within bronchi and bronchioles, a mixed inflammatory infiltrate was observed alongside epithelial degeneration and hyperplasia (Fig. 3d). Moreover, animals exhibited prominent vascular alterations, including endothelial hypertrophy, endothelialitis, mural and perivascular inflammatory cell infiltration, compromised vascular wall integrity and perivascular oedema (Fig. 3e). mRNA/LD MVA-ST–vaccinated hamsters also revealed histopathological findings, but overall substantially less extensive alveolar, bronchial/bronchiolar and vascular lesions (Fig. 3b).

Semiquantitative scoring of airway, vascular and alveolar lesions confirmed these results with significant reduction for most parameters in mRNA/LD MVA-ST–vaccinated animals compared to the control-vaccinated group (Fig. 3f–i).

### HD MVA-ST boost confers robust protective efficacy following mRNA or AdV priming

**Fig. 4.**
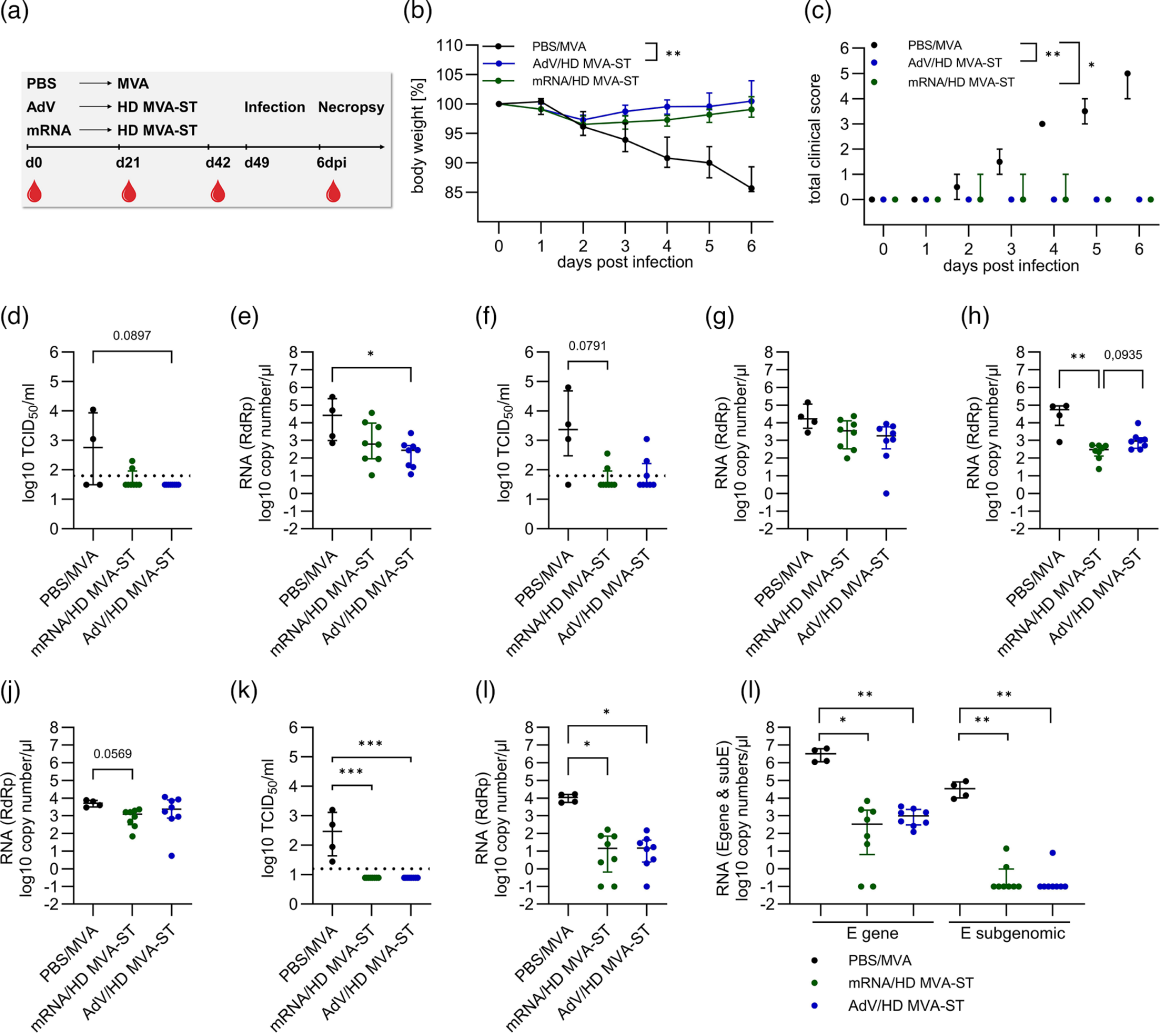
HD MVA-ST booster confers robust protection against SARS-CoV-2 infection following mRNA or AdV priming. (**a**) Groups of hamsters (*n*=4 for PBS/MVA, *n*=8 for mRNA/HD MVA-ST and *n*=8 for AdV/HD MVA-ST) were immunized first with a dose of 12 µg mRNA or a dosage of 1×10^9^ VP for the AdV and boosted 21 days later with 10^8^ p.f.u. (HD) MVA-ST using the intramuscular route. Twenty-eight days later, all animals were challenged with 1×10^4^ TCID_50_ SARS-CoV-2 Bav Pat1 isolate via the intranasal route. (**b**) Body weight was monitored daily. (**c**) Clinical condition was assessed using the clinical scoring system described in the Methods section. (**d, e**) Nasal and (**f, g**) oropharyngeal swabs collected on day 3 post-challenge were analysed for titres of infectious SARS-CoV-2 using TCID_50_ per gram lung tissue and copies of genomic RNA by RdRp qRT-PCR. (**h**) Nasal and (**i**) oropharyngeal swabs collected on day 6 post-challenge were analysed for copies of genomic RNA by RdRp qRT-PCR. (**j–m**) Lung tissues were harvested on day 6 post-challenge and analysed for (**j**) infectious SARS-CoV-2 titres (TCID_50_ per gramme lung tissue) and copies of genomic RNA by (**k**) RdRp qRT-PCR and (**l**) E-gene qRT-PCR and subE qRT-PCR. Differences between the groups were analysed, determining the area under the curve (AUC) (**b, c**) prior to analysis with the Kruskal–Wallis test combined with Dunn’s multiple comparisons test (**b–f**). Data are presented as median values with interquartile range. The asterisks represent statistically significant differences between two groups: *P<0.05, **P<0.01 and ***P<0.001. Exact *P*-values are shown for comparisons with a trend toward significance (0.05≤*P*<0.10). Non-significant differences outside this range are not indicated.

As established before, male Syrian hamsters were vaccinated in a heterologous prime–boost schedule, receiving either 12 µg mRNA or 1×10^9^ VP AdV via intramuscular injection into the left hind leg as prime immunization. Three weeks later, hamsters were boosted with a HD of 1×10^8^ p.f.u. (HD) MVA-ST. PBS primed and MVA boosted (1×10^8^ p.f.u. of empty MVA vector) served as the control group (Fig. 4a). No weight loss or adverse reactions were detected in any group over the 42-day monitoring period before challenge infection (Fig. S2A).

After intranasal infection with 1×10^4^ TCID_50_ SARS-CoV-2 (isolate BaVPat1) on day 49 after the vaccination, all hamsters lost ~2–5% of their body weight 2 dpi. Continued weight loss was seen in the control group, whereas hamsters of the mRNA/HD MVA-ST– and AdV/HD MVA-ST–vaccinated groups did not lose any more weight. The PBS/MVA-vaccinated control hamsters continued to show progressive weight loss until 6 dpi, losing ~15% of their original body weight, as measured on the day of the challenge (Fig. 4b).

Symptoms associated with SARS-CoV-2 infection started to develop 2 dpi in control-vaccinated hamsters and increased in severity until 6 dpi, reaching a cumulative clinical score of 6, corresponding to significant clinical disease. This included weight loss, piloerection and laboured breathing. AdV/HD MVA-ST–vaccinated hamsters showed no signs of clinical disease over the 6-day infection period. Single animals of the mRNA/HD MVA-ST–vaccinated hamsters presented with minimal weight loss on days 2–4 post-infection, resulting in a maximum clinical score of 1 (Fig. 4c).

At 3 dpi, titres of infectious SARS-CoV-2 could be detected in the nasal swabs of the PBS/MVA hamsters with a median of 5.8×10^2^ TCID_50_. Viral shedding from the upper respiratory tract was significantly lower in the mRNA/HD-MVA-ST– and AdV/HD MVA-ST–vaccinated hamsters with no detectable titres in 6/8 animals of the mRNA/HD MVA-ST group and all animals of the AdV/HD MVA-ST group (Fig. 4d). Evaluation of RdRp levels in nasal swabs taken 3 dpi confirmed significantly higher loads in the PBS/MVA control group (median 2.6×10^4^ RNA copy numbers/μl) compared to the mRNA/HD MVA-ST group (median of 6.3×10^2^ RNA copy numbers/μl) and the AdV/HD MVA-ST group (median 2.8×10^2^ RNA copy numbers/μl; Fig. 4e). Overall, levels of viral shedding in both HD MVA-ST–boosted groups were substantially lower than in the LD MVA-ST–boosted group.

TCID_50_ of oropharyngeal swabs taken 3 dpi showed high titres of infectious SARS-CoV-2 in the PBS/MVA animals (median 2.3×10^3^ TCID_50_). Again, substantially lower titres of infectious SARS-CoV-2 were detected in the upper respiratory tract of mRNA/HD MVA-ST– and AdV/HD MVA-ST–vaccinated hamsters (6/8 below detection limit for mRNA/HD MVA-ST and 5/8 below detection limit for AdV/HD MVA-ST; Fig. 4f). This has also been confirmed by the analysis of viral RNA in oropharyngeal swabs, as measured by RdRp levels with a median of 1.7×10^4^ RNA copy numbers/μl for PBS/MVA, median of 3.5×10^3^ RNA copy numbers/μl for mRNA/HD MVA-ST and a median of 1.8×10^3^ RNA copy numbers/μl for AdV/HD MVA-ST (Fig. 4g). Again, the levels of viral shedding from the oropharyngeal swabs in both HD MVA-ST–boosted groups were substantially lower than in the LD MVA-ST–boosted group with the most apparent difference in TCID_50_ titres from oropharyngeal swabs taken 3 dpi (*P*=0.0879).

As established before, all animals were sacrificed 6 dpi. We collected nasal and oropharyngeal swabs as well as blood and organ samples, including lung, spleen and brain from all hamsters.

Nasal swab RdRp levels on 6 dpi were highest in the control hamsters (median 5.5×10^4^ RNA copy numbers/μl), significantly lower in the mRNA/HD MVA-ST hamsters (median 3.1×10^2^ RNA copy numbers/μl) and reduced in the AdV/HD MVA-ST hamsters (median 9.3×10^2^ RNA copy numbers/μl; Fig. 4h).

RdRp levels in oropharyngeal swabs taken on 6 dpi showed a median of 5.2×10^3^ RNA copy numbers/μl for PBS/MVA, median of 1.3×10^3^ RNA copy numbers/μl for mRNA/HD MVA-ST and median of 2.4×10^3^ RNA copy numbers/μl for AdV/HD MVA-ST (Fig. 4i).

TCID_50_ of lung homogenates revealed substantial titres of infectious SARS-CoV-2 (median 2.9×10^2^ TCID_50_) in the control-vaccinated animals. No titres of infectious SARS-CoV-2 were detectable in the lungs of both vaccination groups (Fig. 4j).

RdRp levels in the lung were significantly lower in both vaccinated groups compared to the control group (median 1.1×10^4^ RNA copy numbers/μl for PBS/MVA, median 1.4×10^1^ RNA copy numbers/μl for mRNA/HD MVA-ST and median 1.5×10^1^ RNA copy numbers/μl for AdV/HD MVA-ST; Fig. 4k). RT-qPCR targeting the E gene and subE also showed substantial levels in the PBS/MVA control hamsters (median 3.2×10^6^ RNA copy numbers/μl for E and median 3.4×10^4^ RNA copy numbers/μl for subE). The mRNA/HD MVA-ST–vaccinated hamsters mounted significantly lower median copy numbers of 3.4×10^2^ RNA copy numbers/μl for E and no detectable titres in 6/8 animals for subE. Levels were also significantly lower in AdV/HD MVA-ST–vaccinated hamsters for E with a median of 9.8×10^2^ RNA copy numbers/μl and not detectable titres for subE in 7/8 animals (Fig. 4l).

Evaluation of brain tissue samples for RdRp copy numbers revealed significantly higher levels in the control group (median 2.2×10^1^ RNA copy numbers/μl) compared to the mRNA/HD MVA-ST (median 0.1×10^0^ RNA copy numbers/μl) and AdV/HD MVA-ST groups (median 0.2×10^0^ RNA copy numbers/μl; Fig. S2B). E gene copy numbers showed the highest median value for PBS/MVA (median 4.1×10^3^ RNA copy numbers/μl) and similar median values for mRNA/HD MVA-ST (median 8.3×10^1^ RNA copy numbers/μl) and AdV/HD MVA-ST (median 8.2×10^1^ RNA copy numbers/μl) and significantly different levels between the groups for subE with a median titre of 3.6×10^1^ RNA copy numbers/μl in the control group and no detectable titres in both immunized groups (Fig. S2C).

### HD MVA-ST boost induced a robust activation of SARS-CoV-2-neutralizing antibodies and IFN-*γ*-producing T cells

**Fig. 5.**
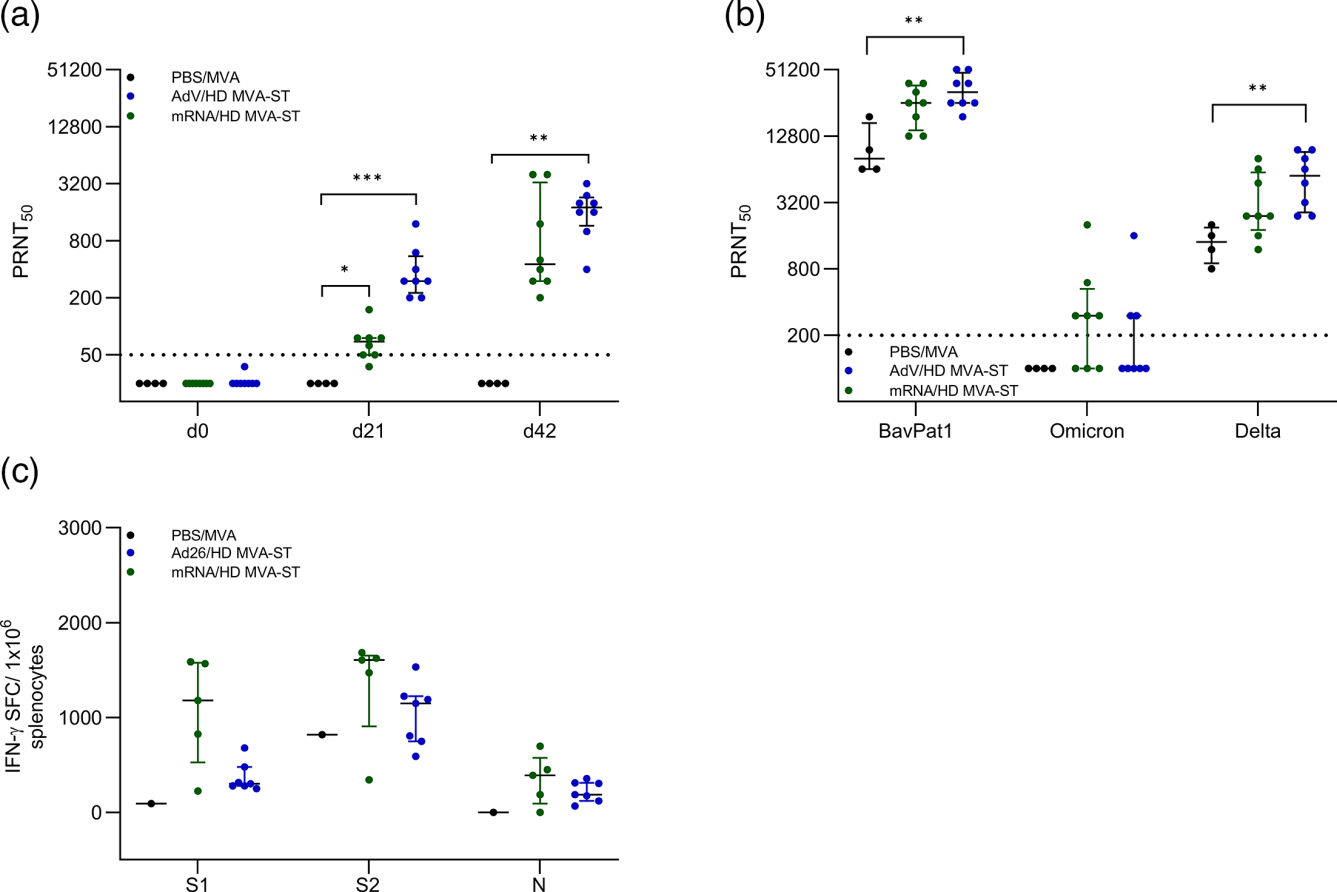
HD MVA-ST booster induces SARS-CoV-2-specific antibody and T cell responses. (**a**) Sera from mRNA/HD MVA-ST or AdV/HD MVA-ST–vaccinated or PBS/MVA-injected hamsters were prepared on days 0, 21 and 42 after vaccination and analysed for SARS-CoV-2 BavPat1–neutralizing antibodies by PRNT_50_. (**b**) Sera from day 6 after challenge infection were evaluated for neutralizing antibodies against three SARS-CoV-2 variants: BavPat1, Omicron and Delta. (**c**) Splenocytes were collected and prepared on day 6 after SARS-CoV-2 BaVPat1 infection and stimulated with pools of overlapping peptides comprising the S1 and S2 subunit of SARS-CoV-2 S and N proteins and tested using ELISpot assay. Differences between the groups were analysed by the Kruskal–Wallis test combined with Dunn’s multiple comparisons test (**a–c**). Dotted lines indicate the respective detection limit for PRNT_50_ (**a, b**). Data are presented as median values with interquartile range. The asterisks represent statistically significant differences between two groups: *P<0.05, **P<0.01 and ***P<0.001. Exact *P*-values are shown for comparisons with a trend toward significance (0.05≤*P*<0.10). Non-significant differences outside this range are not indicated.

We did not detect SARS-CoV-2-specific titres of neutralizing antibodies in the PBS/MVA control hamsters prior to SARS-CoV-2 challenge infection. In contrast, hamsters of the two immunized groups developed measurable titres of neutralizing antibodies as early as 3 weeks after the prime immunization. Animals that received mRNA as prime mounted a median PRNT_50_ titre of 1 : 69. AdV primed hamsters mounted a median PRNT_50_ titre of 1 : 300, which was significantly higher compared to the control as well as the mRNA/HD MVA-ST–vaccinated animals. Twenty-one days after the booster application of HD MVA-ST, titres further increased in both vaccination groups (median 1 : 450 for mRNA/HD MVA-ST and median 1 : 1.800 for AdV/HD MVA-ST; Fig. 5a). After challenge infection, substantial levels of neutralizing antibodies were reached against SARS-CoV-2 BavPat1 with a median titre of 1 : 25.600 PRNT_50_ in mRNA/HD MVA-ST–vaccinated hamsters. AdV/HD MVA-ST–vaccinated animals mounted a median titre of 1 : 32.000, which was significantly higher than the control group (median 1 : 8.000). We also detected titres of Omicron-neutralizing antibodies in mRNA/HD MVA-ST–vaccinated hamsters (median of 1 : 300 PRNT_50_). AdV/HD MVA-ST vaccination induced titres of neutralizing antibodies against Omicron, ranging from 1 : 300 to 1 : 1.600 PRNT_50_, while all hamsters of the control group remained below the detection limit. Substantial titres of neutralizing antibodies against the Delta variant were detectable in the mRNA/HD MVA-ST–vaccinated hamsters (median of 1 : 2.400 PNRT_50_) or AdV/HD MVA-ST–vaccinated hamsters (median of 1 : 5.600 PRNT_50_), while titres were significantly lower in the PBS/MVA-vaccinated hamsters (median of 1 : 1.400 PRNT; Fig. 5b).

We evaluated S1-, S2- and N-specific T cell responses in splenocytes *ex vivo* at day 6 post-infection using ELISpot analysis. Hamsters of the control group mounted only marginal numbers of IFN-*γ*-producing cells after stimulation with S1- (median 92 SFC/10^6^ splenocytes) and higher numbers after stimulation with S2- (median 819 SFC/10^6^ splenocytes) specific peptides. No IFN-γ-producing cells could be detected in PBS/MVA animals upon stimulation with N-specific peptides. mRNA/HD MVA-ST–vaccinated hamsters mounted higher numbers after stimulation with S1-, S2- and N-specific peptides (median 1.179 SFC/10^6^ splenocytes for S1, median 1.606 SFC/10^6^ splenocytes for S2 and median 391 SFC/10^6^ splenocytes for N). AdV/HD MVA-ST hamsters also mounted higher levels of specific T cells (median 301 SFC/10^6^ splenocytes for S1, median 1.148 SFC/10^6^ splenocytes for S2 and median 187 SFC/10^6^ splenocytes for N) than the control group (Fig. 5c).

### Pathological evaluation after HD MVA-ST boost

**Fig. 6.**
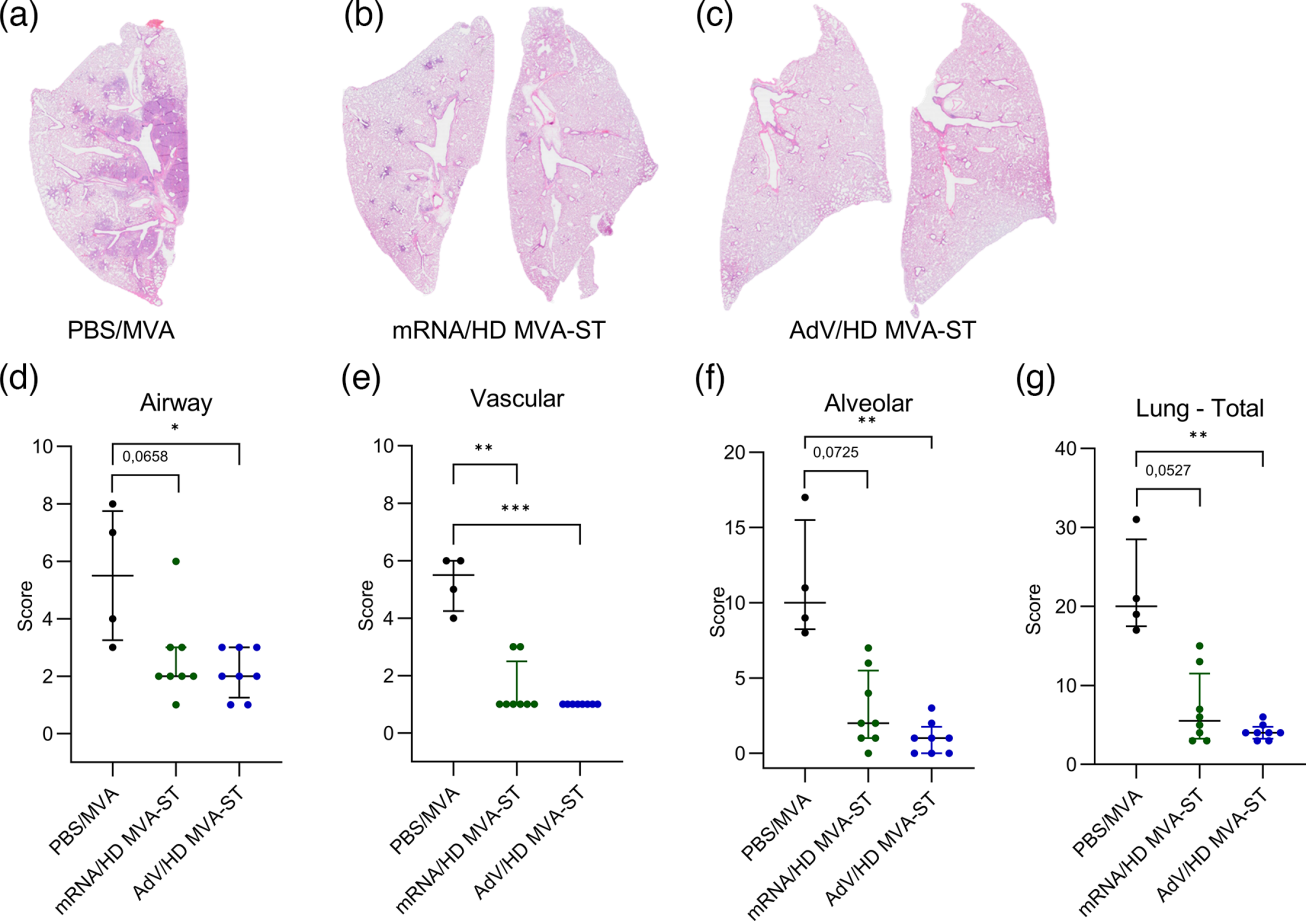
Histopathological lesions in the lungs of SARS-CoV-2 BavPat1–challenged hamsters vaccinated with PBS/MVA, mRNA/HD MVA-ST or AdV/HD MVA-ST. (**a**) Representative overview images of HE-stained lung sections. Lung sections from PBS/MVA control–vaccinated animals show extensive areas of alveolar consolidation with markedly thickened alveolar septae and inflammatory infiltrates, while (**b**) mRNA/HD MVA-ST–vaccinated and (**c**) AdV/HD MVA-ST–vaccinated animals show no alveolar lesions. (**d–g**) Quantification of histopathological lesions by semiquantitative scoring of (**d**) airway lesions score, (**e**) vascular lesions, (**f**) alveolar lesions and (**g**) total scoring of the HE lung. Differences between the groups were analysed by the Kruskal–Wallis test combined with Dunn’s multiple comparisons test (**d–g**). Data are presented as median values with interquartile range. The asterisks represent statistically significant differences between two groups: *P<0.05 and **P<0.01. Exact *P*-values are shown for comparisons with a trend toward significance (0.05≤*P*<0.10). Non-significant differences outside this range are not indicated.

To additionally assess pulmonary pathology in vaccinated and infected animals, we again performed pathohistological evaluations of lung sections, prepared at necropsy on day 6 post-infection, by staining with HE. Hamsters of the PBS/MVA control group exhibited extensive areas of lung consolidation (Fig. 6a). Alveolar damage was marked by the accumulation of neutrophils and mononuclear cells, leading to thickened alveolar septa and the filling of alveolar spaces. Inflammation was accompanied by alveolar epithelial necrosis, fibrin deposition and prominent hyperplasia of type II pneumocytes. Bronchi and bronchioles displayed mixed inflammatory cell infiltration, epithelial degeneration and hyperplasia. Additionally, pronounced vascular pathology was observed, including endothelial hypertrophy, endothelialitis, mural and perivascular infiltrates, compromised vascular wall integrity and perivascular oedema. In contrast, lungs from mRNA/HD MVA-ST–vaccinated hamsters showed minimal to no pathological changes (Fig. 6b). Almost all animals in this group showed only mild inflammatory infiltrates, limited primarily to airways and occasional vessels. Alveolar involvement was rare or minimal, affecting only small lung lobe areas. AdV/HD MVA-ST–vaccinated hamsters also presented very reduced inflammation compared to the control group with minimal inflammatory lesions and regions of consolidation (Fig. 6c).

These results were also evaluated in a semiquantitative scoring system of airway, vascular and alveolar lesions to better assess significant differences between groups (Fig. 6d–g).

### Lung inflammatory responses induced by HD MVA-ST booster

**Fig. 7.**
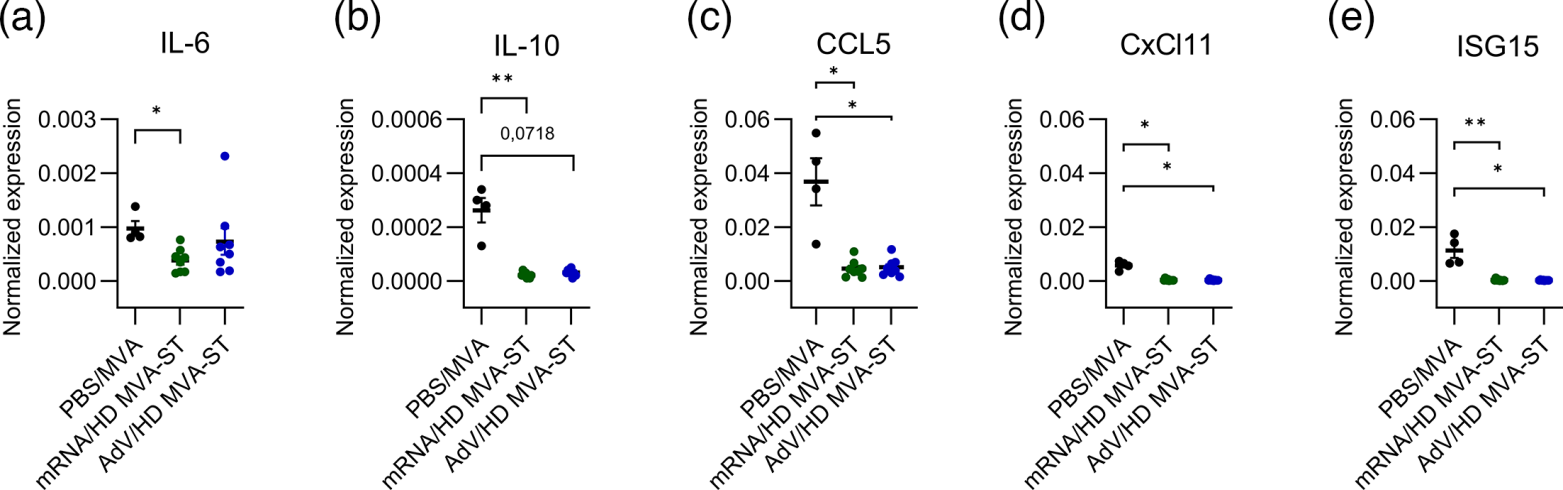
Cytokine expression in the lungs of hamsters after HD MVA-ST boost was analysed by RT-qPCR. Lung tissues were collected from hamsters at day 6 post-challenge, and total RNA was extracted for RT-qPCR analysis. Expression levels of (**a**) IL-6, (**b**) IL-10, (**c**) CCL5, (**d**) CxCl11 and (**e**) ISG15 were measured and normalized to *β*-actin. Differences between the groups were analysed by the Kruskal–Wallis test combined with Dunn’s multiple comparisons test (**a–e**). Data are presented as median values with interquartile range. The asterisks represent statistically significant differences between two groups: *P<0.05 and **P<0.01. Exact *P*-values are shown for comparisons with a trend toward significance (0.05≤*P*<0.10). Non-significant differences outside this range are not indicated.

Different cytokines and the IFN-induced gene ISG15 were characterized in lung tissue by using different RT-qPCR assays. PBS/MVA control animals showed substantial levels of IL-6 expression (median 0.00086 normalized to *β*-actin). mRNA/HD MVA-ST and AdV/HD MVA-ST hamsters mounted lower levels (median of 0.00039 normalized to *β*-actin for mRNA/HD MVA-ST and median of 0.000565 normalized to *β*-actin for AdV/HD MVA-ST; Fig. 7a). The same pattern could be measured for the expression of IL-10 (median 0.00029 normalized to *β*-actin for PBS/MVA, 0.00002 normalized to *β*-actin for mRNA/HD MVA-ST and 0.00003 normalized to *β*-actin for AdV/HD MVA-ST; Fig. 7b). CCL5 expression was significantly reduced in both vaccination groups (median of 0.003935 normalized to *β*-actin for mRNA/HD MVA-ST and median 0.00422 normalized to *β*-actin for AdV/HD MVA-ST) compared to the control animals (median 0.03935 normalized to *β*-actin; Fig. 7c). CxCl11 was also significantly less expressed in both vaccination groups (median 0.000205 normalized to *β*-actin for mRNA/HD MVA-ST and median 0.00021 normalized to *β*-actin for AdV/HD MVA-ST) compared to the PBS/MVA-vaccinated hamsters with a median of 0.00606 normalized to *β*-actin (Fig. 7d). ISG15 expression was significantly higher in the PBS/MVA group (median 0.01051 normalized to *β*-actin) compared to the vaccinated animals (median 0.00019 normalized to *β*-actin for mRNA/HD MVA-ST and median 0.00023 normalized to *β*-actin for AdV/HD MVA-ST; Fig. 7e).

## Discussion

During the COVID-19 pandemic, the rapid availability of several licensed vaccines played a critical role in controlling SARS-CoV-2 infections in human populations [23]. While homologous vaccination regimens are now well established, heterologous combinations have been widely implemented across many countries due to practical considerations such as vaccine supply shortages, changes in safety recommendations (e.g. vector-based vaccines) or the need to optimize immune responses [24]. However, specific data on the efficacy and immunogenicity of heterologous strategies remains limited.

In this study, we evaluated an MVA-based vaccine expressing the prefusion-stabilized version of the SARS-CoV-2 S protein (MVA-ST) as a heterologous boost vaccination following adenovirus or mRNA priming in the Syrian hamster model. The Syrian golden hamster model has proven highly valuable for studying SARS-CoV-2 infection, as it mirrors several key aspects of COVID-19 in humans, including viral replication dynamics, lung pathology and innate immune activation [25,27]. Importantly, hamsters also mount robust humoral immune responses, including the generation of neutralizing antibodies, which closely resemble those observed in humans [28]. This makes the model particularly useful for evaluating vaccine efficacy and antibody-based therapies. The antibody responses observed in hamsters have been shown to correlate well with protective immunity in humans, supporting the translational relevance of our findings to human immune responses against SARS-CoV-2 [29].

In this study, we show that for COVID-19, the heterologous regimen induced enhanced immunogenicity compared to homologous MVA-ST vaccination strategies from our previous studies [16], resulting in robust protection against SARS-CoV-2 infection. These previous preclinical studies already demonstrated that two doses of the MVA-ST vaccine provide safety, immunogenicity and protection in Syrian hamsters [16,20]. While the comparison to our previous homologous vaccination study was based on data generated in an earlier study, all experiments were conducted under strictly standardized conditions, including the same animal cohort sources, vaccination and challenge protocols and immunological assays, which support the validity of the comparison. Of course, a perfect comparison would require a direct head-to-head experiment conducted within the same study. While this was not feasible, the high level of standardization across both studies allows for a meaningful comparison, although some limitations remain and should be considered when interpreting the results.

The robust immunogenicity of homologous MVA-ST vaccination has been further confirmed in a phase Ib clinical study in humans. Additionally, the phase Ib study identified a positive effect of a heterologous vaccination regimen using the MVA-ST as a boost after mRNA prime vaccination. Of note, this positive effect has also been seen for a lower dose boost using 1×10^7^ p.f.u. MVA-ST (LD) [17].

Given the potential of dose-sparing strategies, we first assessed the protective efficacy of an LD heterologous boost vaccination in the hamster challenge model after mRNA prime vaccination. The mRNA-prime/LD MVA-ST boost immunization regimen led to higher neutralizing antibody titres than the homologous MVA-ST vaccination regimen from our previous studies [16]. It is important to acknowledge the inherent limitation posed by the indirect comparison with the homologous MVA-ST vaccination study; however, given that the experimental conditions were consistent across studies, as outlined above, a comparison remains valid despite this constraint. mRNA/LD MVA-ST–vaccinated hamsters exhibited significantly reduced signs of clinical disease following intranasal SARS-CoV-2 challenge compared to the control-vaccinated hamsters, as reflected in clinical scoring. The favourable clinical disease outcome was also reflected by complete viral clearance in the lungs of the mRNA/LD MVA-ST–vaccinated animals 6 dpi. However, despite this favourable clinical outcome, the protective efficacy in terms of viral clearance was slightly reduced as evidenced by SARS-CoV-2 shedding from the upper respiratory tract. Specifically, viral titres from nasal and oropharyngeal swabs were higher compared to those in animals receiving a homologous MVA-ST vaccination from previous studies [16]. Post-challenge, neutralizing antibody titres in the heterologously vaccinated group were not significantly different from those of control animals, which might explain the marginal viral shedding from the upper respiratory tract. The lower titres of neutralizing antibodies could indicate suboptimal B cell affinity maturation in the weeks following the LD booster, potentially limiting sustained antibody production [30,31]. This observation aligns with results from the phase Ib clinical study in humans [17], where a late boost effect was not observed, unlike in other homologous MVA vaccination approaches in humans [32]. Such differences may be explained by a suboptimal B cell maturation with the LD MVA-ST due to the phenomenon of baseline dependency, previously described in observational studies of booster responses across different pathogens [33]. Currently, data on heterologous vaccination regimens using an LD boost are rare. Most heterologous regimens use equivalent booster doses as in homologous protocols [34], and no similar effect after an LD boost has been reported in the context of other vaccination platforms [35]. In contrast, some homologous vaccination strategies have explored dose sparing with promising results. This has been demonstrated for another MVA-based COVID-19 vaccine expressing a non-stabilized S-protein (MVA-S) [36], which showed strong neutralizing antibody responses when administered as an LD intranasal boost vaccination following intramuscular priming [36,37]. Similar immunogenicity at this dose was also reported in a mouse model evaluating MVA-based vaccines against Lassa virus [38]. However, since these mouse studies lacked a challenge infection, conclusions about immune responses and the outcome of protection are hampered. Altogether, our findings suggest that while heterologous LD-MVA-ST boost after mRNA priming provides strong initial protection and clinical benefit, it may result in suboptimal viral clearance and antibody maturation. Further studies are needed to dissect the underlying immunological mechanisms, particularly B cell dynamics and affinity maturation, following LD heterologous vaccination.

In humans, breakthrough infections were reported during the early phase of the pandemic, particularly in individuals who received mRNA prime vaccination followed by suboptimal booster doses of mRNA vaccine due to dose-sparing strategies [39]. These breakthrough infections were typically associated with mild clinical symptoms. Interestingly, our study demonstrated that an HD (10^8^ p.f.u.) MVA-ST boost after mRNA prime robustly protected hamsters against intranasal SARS-CoV-2 challenge as evidenced by the lack of viral shedding from the upper respiratory tract and no viral titres in the lungs. In addition, no clinical disease symptoms were detected in the mRNA/HD MVA-ST–vaccinated animals. This robust protection could be associated with strong activation of nAb before and after the SARS-CoV-2 infection.

Interestingly, post-challenge nAb titres were significantly higher in animals receiving the HD MVA-ST boost compared to those given the LD MVA-ST boost. This suggests that a higher booster dose may promote more effective B cell affinity maturation following infection, resulting in significantly higher titres of nAb. We also examined a heterologous regimen with a licensed AdV SARS-CoV-2 vaccine for priming, followed by an HD MVA-ST boost. Again, this approach conferred robust protection, with strong induction of both nAbs and T cell responses, comparable to the mRNA prime/HD MVA-ST boost strategy. Notably, in the indirect comparison with the previous homologous MVA-ST vaccination, both heterologous regimens induced stronger immune responses [16,20]. These results align with prior preclinical studies that demonstrated superior immunogenicity and efficacy of heterologous COVID-19 vaccination strategies [40]. They also corroborate findings from human studies, such as that by Barros-Martins *et al*. [12], which showed stronger antibody responses in individuals receiving heterologous ChAdOx1/BNT162b2 regimens compared to homologous ChAdOx1 vaccination.

A key aim of our study was to evaluate the protective efficacy of heterologous vaccination strategies. These strategies have already been tested for immunogenicity in a phase Ib human trial. Our findings confirm that boosting with HD MVA-ST enhances protection, regardless of whether the initial prime was mRNA- or AdV-based. After the challenge, both vaccination regimens induced strong nAb responses. However, S-specific T cell responses were lower in the AdV-prime group. This suggests that nAbs may play a more dominant role in protection in this setting. Results from our LD MVA-ST boost experiment support this idea. Although T cell responses were similar in both the LD and HD MVA-ST boost groups, the LD group showed slightly reduced protection and significantly lower nAb titres. This further highlights the importance of nAbs in mediating protective immunity. This is also in line with results from a multitude of previous studies, which identified that the titres of nAb positively correlated with the outcome of protection [41,43]. Of note, a recent study also confirmed the key role for nAb after heterologous prime–boost vaccinations in humans. Here, heterologous prime–boost vaccination using ChAdOx1 followed by an mRNA-1273 booster dramatically enhanced serum neutralizing antibody titres and memory B cell responses against multiple SARS-CoV-2 variants, compared to homologous ChAdOx1 boosting [44]. These results further support the use of heterologous prime–boost vaccinations. So far, no clinical data are available on the correlation of the immune responses induced by AdV or mRNA prime vaccination and MVA-ST boost with the outcome of protection. Thus, the results from our study provide first insights into the usability of MVA-ST as a boost vaccine for COVID-19. Based on our data, we conclude that combining MVA-based vaccines with other platforms, such as mRNA or AdV, may be advantageous in humans. This has already been confirmed for the licensed EBOV vaccine regimen Mvabea (BN, Denmark). Mvabea includes an AdV-EBOV prime followed by an MVA-EBOV boost. That combination is advantageous due to the capacity of MVA to strongly boost both T cell and antibody responses without being compromised by pre-existing vector immunity induced by AdV-based vaccines. Likewise, in the context of mRNA priming, protein-based boosters may offer the added benefit of inducing strong and durable T cell responses, complementing the typically antibody-dominant profile of mRNA vaccines.

Using an MVA-based vaccine as a booster in the heterologous vaccination strategy offers several advantages. Notably, it reduces the need for large-scale production of MVA-based vaccines, a process that remains technically complex and more resource-intensive than other platforms such as mRNA [45]. By reserving the MVA-based vaccine for boosting, its potent immunogenicity can be effectively harnessed while minimizing manufacturing burdens and supply constraints. This approach also focuses on the favourable safety profile and compatibility of the MVA platform with a wide range of vaccines that can be used for priming, including DNA, mRNA and AdVs [17,46].

The safety and compatibility have been further confirmed in our study by the results from pathology. Pathological analysis in the hamster model showed no signs of lung inflammation following heterologous prime–boost vaccination, highlighting the safety of this approach. In addition, animals that received a HD MVA-ST boost showed a marked reduction in the expression of key inflammatory and IFN-stimulated genes following SARS-CoV-2 infection. These included IL-6, IL-10, CCL5, CXCL11 and ISG15. These molecules are involved in both antiviral responses and immunopathology. IL-6 and IL-10 help regulate inflammation by limiting excessive immune activation. CCL5 and CXCL11 recruit T cells and other immune cells to sites of infection. ISG15 amplifies IFN signalling and enhances antiviral defences. Their reduced expression in vaccinated individuals suggests that vaccines help prevent excessive immune activation while preserving effective viral control [47]. Recent studies suggest that heterologous vaccination leads to a more balanced and stronger modulation of inflammatory markers like IL-6, IL-10, CCL5, CXCL11 and ISG15 than homologous approaches [48]. This further supports the use of heterologous prime–boost vaccinations. Our results further support the conclusion that the use of an HD MVA-ST boost provides a promising strategy to improve COVID-19 vaccination strategies with regard to safety, immunogenicity and efficacy. An important aspect for future studies is to evaluate the protective efficacy of the heterologous MVA-ST boost against other SARS-CoV-2 variants. Previous heterologous prime–boost regimens have shown enhanced protection against various SARS-CoV variants, likely due to a broader and more reactive immune response. This effect may also be attributed to the heterologous MVA-ST boost in our study [44]. Supporting this, our *in vitro* data showed strong neutralizing activity against other SARS-CoV-2 variants in sera from heterologous vaccinated hamsters. This also included neutralizing antibodies against the SARS-CoV-2 Delta and Omicron variants. Of note, in the indirect comparison to the previous homologous MVA-ST vaccination study, the titres of heterologous vaccinated hamsters were increased even in the LD-MVA-ST boost. Moreover, given the high prevalence of prior SARS-CoV-2 exposure in the population, the heterologous vaccination strategy examined here may serve as an effective booster to enhance and broaden existing immunity. This is further supported by follow-up analyses showing improved neutralizing activity against multiple variants of concern, underscoring the potential of this approach in diverse immunological backgrounds. These findings suggest that *in vivo* studies assessing the cross-variant protection of this heterologous strategy are promising.

Additionally, the durability of immunity conferred by MVA-based boosters must be assessed in long-term studies. The longevity of SARS-CoV-2-specific immune responses after heterologous vaccination should be further investigated. A known limitation of mRNA vaccines is that the duration of humoral immunity can vary, influenced by factors such as the antigen, vaccine formulation, dosing schedule and host. As a result, repeated booster doses are often needed to maintain protective antibody levels [49]. Similarly, AdV-based vaccines face challenges with durability due to anti-vector immunity, which can reduce the effectiveness of repeated homologous boosting [50]. Thus, heterologous vaccination strategies, whether mRNA or AdV prime, might benefit from a heterologous booster to enhance the longevity and durability of both humoral and cellular immune responses. This has been confirmed in several previous studies. A recent study demonstrated that heterologous prime–boost vaccination (mRNA prime/AdV boost) significantly improves the durability of immune responses [51]. A positive effect on the longevity of specific immune responses has also been demonstrated for a heterologous human immunodeficiency virus (HIV)-DNA prime/HIV-MVA boost regimen. Moreover, Mvabea, the licensed Ebola vaccine, also consists of a heterologous vaccination approach using adenovirus prime and MVA-boost vaccination. First follow-up results indicated a superior durability of EBOV-specific immune responses [52]. This induced long-lasting antibody and T cell responses, maintained for at least 3 years. A late third HIV-MVA dose effectively boosted these responses, confirming the durability and recall capacity of heterologous vaccination strategies [53]. So for this, a long-time follow-up of the immune responses in the heterologously vaccinated and challenged hamster is interesting.

A limitation of our study is the lack of a direct head-to-head comparison with homologous prime–boost regimens using mRNA and AdV platforms. Although limitations inherent to indirect comparisons persist, the rigorous standardization of experimental conditions across both studies supports a meaningful and valid comparison. This is exemplified by our approach to the homologous MVA-ST vaccination regimen, where results were compared to previously established data generated in our laboratory under similar, highly controlled conditions [16]. However, the absence of direct comparisons with the homologous AdV or mRNA vaccination regimens limits the interpretability of relative efficacy. However, a direct comparison to the results from the homologous mRNA and AdV vaccination approaches is difficult since we have not established that in our setting. Thus, for the interpretation, we focused on the effect and usability of the MVA-ST boost vaccination compared to our homologous vaccination approaches. MVA-based boost vaccination in the context of heterologous vaccination regimens has already demonstrated safety, strong immunogenicity and protection in several preclinical models [54]. In addition, it is part of a licensed human vaccination strategy [55]. Our findings further support the use of MVA-based vaccines as booster candidates. In conclusion, we provide the first *in vivo* evidence that an MVA-ST booster following AdV or mRNA priming confers robust protection, characterized by high neutralizing antibody titres and strong T cell responses. Even the LD MVA-ST boost vaccination after mRNA prime protected from severe disease. However, since the shedding from the upper respiratory tract was lower in the HD MVA-ST than the LD MVA-ST boost, we prefer to continue with the HD MVA-ST boost. These findings justify further clinical evaluation in humans. They may also inform future heterologous prime–boost strategies for other infectious diseases, including influenza A virus, which remains a global threat due to its potential for sudden outbreaks and the emergence of novel variants [56]. Together, these findings underscore the strong potential of MVA as a heterologous booster not only to enhance and broaden the immunogenicity and protective efficacy of existing COVID-19 vaccines but also to strengthen future vaccination strategies against emerging infectious diseases.

## Supplementary material

10.1099/jgv.0.002180 Uncited Supplementary Material 1.

## Abbreviations

AdV: adenoviral vector
BSL: biosafety level
COVID-19: coronavirus disease 2019
DMEM: Dulbecco’s Modified Eagle Medium
dpi: days post-infection
EBOV: Ebola virus
ELISpot: enzyme-linked ImmunoSpot
HD: high dose
HE: haematoxylin and eosin
HIV: human immunodeficiency virus
LD: low dose
MVA: modified vaccinia virus Ankara
PRNT_50_: plaque reduction neutralization test 50
RdRp: RNA-dependent RNA polymerase
RT-qPCR: reverse transcription quantitative real-time PCR
S1: subunit 1
S2: subunit 2
SARS-CoV-2: severe acute respiratory syndrome coronavirus 2
SFC: spot-forming cell
VP: viral particle
