# Supplementary material for: Strong immunogenicity and protection against SARS-CoV-2 in hamsters induced by heterologous boost vaccination with an MVA-based COVID-19 vaccine candidate
Source: J Gen Virol. 2025 Nov 19;106(11):002180. doi: 10.1099/jgv.0.002180 (PMC12629099; doi:10.1099/jgv.0.002180)
Supplement: Uncited Supplementary Material 1. [file jgv-106-02180-s001.pdf]

Supplementary Materials for

Strong Immunogenicity and Protection Against SARS-CoV-2 in Hamsters Induced by Heterologous Boost Vaccination with an MVA-Based COVID-19 Vaccine Candidate

Ohrnberger et al.

*Corresponding author. Email: Asisa.Volz@tiho-hannover.de

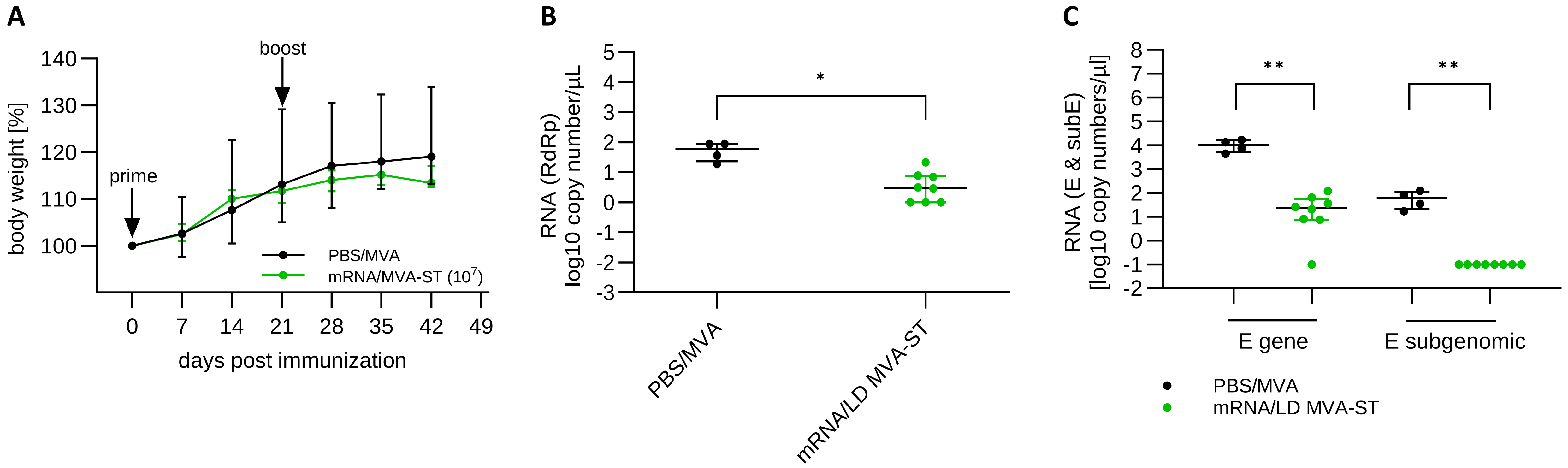

Figure S1: Safety of heterologous mRNA/LD MVA-ST vaccination and viral load in brains after SARS-CoV-2 infection.
(A) Groups of hamsters (n = 4 for PBS/MVA, n = 8 for mRNA/LD MVA-ST) were immunized first with a dose of 12 µg mRNA and boosted 21 days later with LD 10^7 PFU MVA-ST using the intramuscular route. Body weight was monitored over the 42 days immunization period. All hamsters were infected with 1×10^4 $TCID_{50}$ SARS-CoV-2 (BavPat1) intranasally on day 49. Viral load was assessed by (B) RdRp and (C) E and subgenomic E RT-qPCR analysis of brain tissue. Differences between the groups were analyzed, determining the area under the curve (AUC) (A) prior to analysis with Kolmogorov–Smirnov test (A-C). Data points indicate median and interquartile range within the groups. Asterisks represent statistically significant differences between two groups: * $p < 0.05$, ** $p < 0.01$

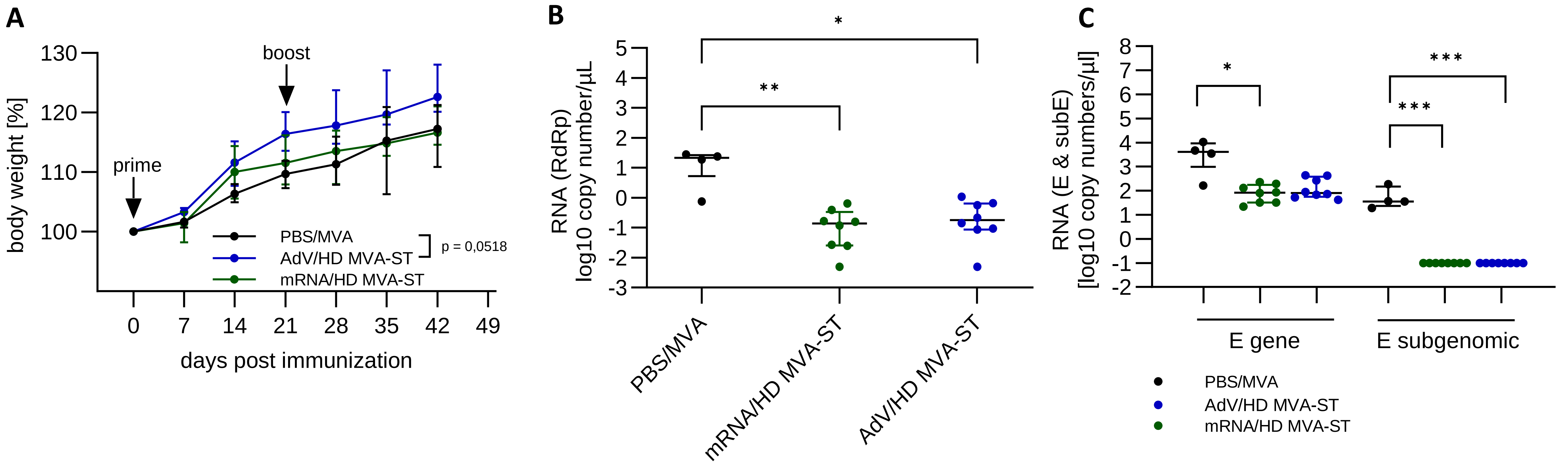

Figure S2: Safety of heterologous HD MVA-ST Boost vaccination and viral load in brains after SARS-CoV-2 infection.
(A) Groups of hamsters (n = 4 for PBS/MVA, n = 8 for mRNA/HD MVA-ST, n = 8 for AdV/HD MVA-ST) were immunized first with a dose of 12 µg mRNA or 1 x 10^9 VP AdV and boosted 21 days later with HD 10^8 PFU MVA-ST using the intramuscular route. Body weight was monitored over the 42 days immunization period. All hamsters were infected with 1×10^4 $TCID_{50}$ SARS-CoV-2 (BavPat1) SARS-CoV-2 intranasally on day 49. Viral load was assessed by (B) RdRp and (C) E and subgenomic E RT-qPCR analysis of brain tissue. Differences between the groups were analyzed, determining the area under the curve (AUC) (A) prior to analysis with Kruskal-Wallis test combined with Dunn's multiple comparisons test (A-C). Data points indicate median values and interquartile range within the groups. Asterisks represent statistically significant differences between two groups: * $p < 0.05$, ** $p < 0.01$, *** $p < 0.001$. Exact p-values are shown for comparisons with a trend toward significance ($0.05 \le p < 0.10$). Non-significant differences outside this range are not indicated.
